# *Mycoplasma pneumoniae* may cause dyspnoea and hospitalisations in young healthy adults

**DOI:** 10.1007/s10096-021-04171-z

**Published:** 2021-02-03

**Authors:** Riku Metsälä, Solja Ala-Korpi, Juha Rannikko, Merja Helminen, Marjo Renko

**Affiliations:** 1 Faculty of Medicine and Health Technology, Tampere University, Tampere, Finland; 2 Tampere University Hospital, Tampere, Finland; 3 Tampere Center for Child Health Research, Tampere, Finland; 4 Department of Pediatrics, University of Eastern Finland and Kuopio University Hospital, Box 100, 70029 Kuopio, Finland; 5 PEDEGO Research Unit, University of Oulu, Oulu, Finland

**Keywords:** Community-acquired pneumonia, Shortness of breath, Respiratory infection, Clinical features, *Mycoplasma pneumoniae*

## Abstract

Polymerase chain reaction (PCR)-based diagnostics for *Mycoplasma pneumoniae* (*M. pneumoniae*) from the respiratory tract has become widely available, but the interpretation of the results remains unclear. *M. pneumoniae* has been suggested to cause mainly mild and self-limiting infections or asymptomatic carriage. However, systematic analyses of the association between PCR results and clinical findings are scarce. This study aimed to clarify the clinical features of PCR-positive *M. pneumoniae* infections in a hospital setting. We reviewed 103 PCR-positive patients cared for in a university hospital during a 3-year period. Data on age, sex, health condition, acute symptoms, other pathogens found, laboratory and X-ray results and treatments were collected. Over 85% of the patients had a triad of typical symptoms: fever, cough and shortness of breath. Symptoms in the upper respiratory tract were rare. In 91% of the cases, *M. pneumoniae* was the only pathogen found. The highest incidence was found in the age group of 30–40 years, and 68% of the patients did not have any underlying diseases. Most patients were initially empirically treated with beta-lactam antibiotics and needed 2–4 changes in their treatment. Only 6% were discharged without an antibiotic effective against *M. pneumoniae*. This study shows that *M. pneumoniae* often led to hospitalisation and that patients needed appropriate antimicrobial treatment to recover. Mixed infections were rare, and situations that could be interpreted as carriage did not occur.

## Introduction

*Mycoplasma pneumoniae* (*M. pneumoniae*, mycoplasma) is one of the smallest bacteria in terms of cellular dimensions and genome [1]. *M. pneumoniae* typically infects children and young adults [2], and it has been suggested to mainly cause mild and self-limiting respiratory tract infections usually managed by primary health care [3]. *M. pneumoniae* is also capable of causing community-acquired pneumonias (CAP) and epidemics in schools, camps, military bases and other communities [4, 5]. Although the majority of mycoplasma infections are mild, severe respiratory manifestations may require intensive care unit admissions [6]. Extrapulmonary manifestations, such as Stevens-Johnson syndrome, pericarditis or encephalitis, are also possible [7].

In the past, the lack of quick and efficient microbiological methods made the clinical diagnosis of *M. pneumonia* infections challenging [8]. Although nucleic acid tests, such as polymerase chain reaction (PCR), have become widely available, the role of *M. pneumoniae* in the aetiology of respiratory infections remains unclear. Studies using PCR tests have shown that *M. pneumoniae* is carried at relatively high rates in the respiratory tract of healthy children [1]. Conversely, infection may lead to intensive care in adults [3]*.* Randomised trials on treatment have not been published [9], and it has been argued whether *M. pneumoniae* is a real pathogen requiring treatment.

To clarify the role of *M. pneumoniae* in respiratory infections, we evaluated the clinical course of PCR-positive *M. pneumoniae* infections in a hospital setting. We also assessed the frequency of spontaneous recovery and the proportion of mixed infections in these mycoplasma PCR-positive patients.

## Materials and methods

We performed a retrospective case analysis of *M. pneumoniae* PCR*-*positive patients at the Tampere University Hospital, Finland. The Tampere University Hospital serves an area of 530,000 [10] inhabitants as both a secondary and a tertiary referral hospital. Multiplex PCR, which recognises *M. pneumoniae*, *Chlamydia pneumoniae*, *Legionella pneumophila*, *Bordetella pertussis* and *Bordetella parapertussis*, was introduced in March 2014 in our hospital (Seegene Anyplex™ II RB5, South Korea and Bio-Rad CFX96, USA). The results of the test were available for clinicians usually in one working day. We collected data on consecutive patients who had multiplex PCR taken from a nasopharyngeal swab sample between March 2014 and February 2017. The medical records of *M. pneumoniae* PCR-positive patients were reviewed. Only community-acquired infections were included.

We recorded the data on age, sex, underlying diseases, acute symptoms, diagnoses and duration of hospital stay; the highest values of inflammatory parameters during hospital stay (C reactive protein [CRP] and leucocytes); results of all microbial tests available (e.g. blood cultures and tests for respiratory viruses); thoracic X-rays; and antimicrobial treatments. Antimicrobial treatments were classified based on their efficacy against *M. pneumoniae*. Macrolides, doxycycline, tetracyclines and fluoroquinolones were considered effective treatments [4]. Data on acute symptoms, such as fever, cough, rhinitis and shortness of breath, were recorded based on medical records either as the patients’ subjective experience or the clinicians’ assessment. If the symptoms were not mentioned as present or absent, the data were recorded as missing.

The multiplex PCR test for respiratory viruses included adenovirus; influenza A and B viruses; parainfluenza virus types 1–4; rhino-, respiratory syncytial-, boca- and metapneumoviruses; coronavirus types 229E, NL63 and OC43; and enterovirus (Seegene Anyplex™ II RV16, South Korea and Bio-Rad CFX96, USA). Blood cultures were collected in BD BACTEC blood culture bottles and placed in the automated microbial detection system BD BACTEC FX (Franklin Lakes, NJ, USA).

*M. pneumoniae* serology or culture of the swabs was not systematically performed.

In the statistical analyses, we calculated the proportions for the dichotomous variables and the medians and ranges for the continuous variables. The proportion differences between the two groups with 95% confidence intervals were computed with the standard normal deviate test (Stats Direct 3.0). The age distribution of the patients was plotted in 10-year age groups. The time of year of the infections was plotted by summarising the cases in all 3 years in each month.

## Results

**Fig. 1:**
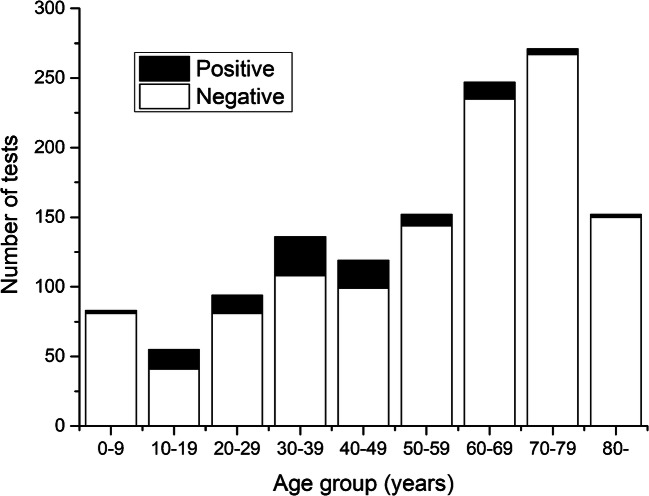
Age distribution of the 103 patients with a positive or negative *M. pneumonia*e PCR tests in a hospital-based setting

**Fig. 2:**
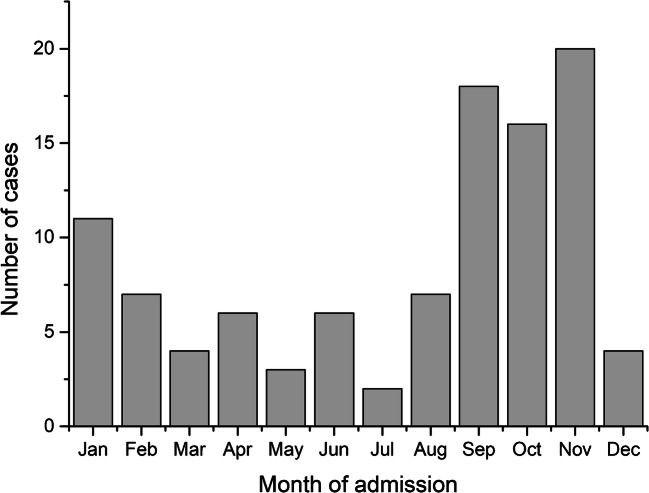
Number of monthly admissions of patients with a positive *M. pneumoniae* PCR test in a hospital-based setting

Respiratory bacterial multiplex PCR was taken from 1,309 patients (1,190 adults and 119 children) during the 3-year study period. *M. pneumoniae* PCR was positive in 103 (8%) cases, the majority of which were males (59, 57%). The median age of the PCR-positive patients was 39 years (range 3.6–87.9) (Table 1). The number of PCR tests taken was the highest in the age groups of 60–80 years and 0–10 years, but the incidence of positive results was the highest in the age group of 30–40 years (Fig. 1). Most of the cases were detected from September to November (Fig. 2). Three patients were discharged from the emergency room, while the remaining patients needed hospital care for a median duration of 5 days (range 1–32 days) (Table 1). Five (5%) patients were admitted to the intensive care unit. Any kind of underlying disease was present in 30 (29%) patients (Table 1). The multiplex PCR test was taken on admission or during the first day of care in 70 (68%) patients.

**Table 1:** Basic characteristics of the 103 patients with a positive *M. pneumonia*e PCR in a hospital-based setting

Characteristic	*N* = 103
Males, *N* (%)	59 (57.3%)
Age (years), mean (SD), [range]	39.9 (17.9), [3.6–87.9]
Fever > 38 °C, *N* (%)	87 (84.5%)
Cough, *N* (%)	99 (99%)
Shortness of breath, *N* (%)	85 (88.5%)
Rhinitis, *N* (%)	14/63 (22.2%)
Max CRP, median [range]	142 [1.9–449]
Max leukocytes, median [range]	11 [1.6–63]
Pneumonia in chest radiograph, *N* (%)	92 (91.1%)
Pleural fluid in chest radiograph, *N* (%)	9 (8.9%)
Length of hospital stay (days), median [range]	5 [0–32]
Underlying diseases, yes (%)	30 (29.1%)
- Asthma, *N* (%)	8 (27%)
- Cardiac disease, *N* (%)	7 (23%)
- Malignancy, *N* (%)	3 (10%)
- Hypertension, *N* (%)	3 (10%)
- COPD, *N* (%)	2 (7%)
- Other, *N* (%) Epilepsy (3), rheumatism (2), HIV (1), anaemia (1), hyponatremia (1), hypothyroidism (1), juvenile idiopathic arthritis (1)	10 (33%)

The most common symptoms were cough in 99 (99%) patients, fever ≥ 38 °C in 87 (85%) and shortness of breath in 85 (89%). Information on the upper respiratory tract symptoms was available in 63 (61%) patients, and 14 (22%) of them had rhinitis (Table 1). The acute symptoms did not differ between previously healthy patients and those with underlying diseases (Table 2). Three patients, two children and one adult, had symptoms and signs compatible with Stevens-Johnson syndrome. Thoracic X-ray was performed in 101 (98%) cases, and 94 (91%) images showed signs of pneumonia and 9 (9%) of pleural fluid (Table 1).

**Table 2:** Symptoms of *M. pneumoniae* infection in 73 previously healthy patients and 30 patients with some underlying diseases

	Healthy *N* = 73	Underlying disease *N* = 30	Difference (95% CI)	*P* value
Fever 38 °C, *N* (%)	64 (88%)	23 (77%)	11% (−4 to 30)	0.15
Cough, *N* (%)	71 (97%)	28 (93%)	4% (−4 to 19)	0.33
Shortness of breath, *N* (%)	59 (81%)	26 (87%)	−5% (−20 to 12)	0.42
Rhinitis, *N* (%)	9/45 (20%)	5/18 (28%)	−8% (−33 to 13)	0.36

None of the patients had other bacteria aside from *M. pneumoniae* in the multiplex PCR. Blood culture was performed in 95 (92%) patients and 94 of them were negative. One child had a blood culture positive for *Streptococcus pneumoniae*. Multiplex PCR for respiratory viruses was obtained from 88 (85%) patients, and positive results were found in eight patients, including rhino- (4), boca- (2), corona- (1) and human metapneumoviruses (1). Influenza or respiratory syncytial viruses were not detected.

All 103 patients were treated with antimicrobial agents. In almost all patients (102, 99%), one or more antibiotic changes were made during hospitalisation. Ten (10%) patients received treatment effective against *M. pneumoniae* immediately on admission. However, the second antimicrobial choice was effective against mycoplasma in 50 (49%) patients, the third in 34 (33%) and the fourth in 3 (3%). Overall, 12 (12%) patients were treated with macrolides, 48 (47%) with fluoroquinolones, 21 (20%) with doxy- or tetracycline and 16 (16%) with a combination of effective antimicrobials. Six (6%) patients were discharged without treatment effective against *M. pneumoniae*.

## Discussion

The effect of *M. pneumoniae* as a respiratory pathogen has long been debated. In previous literature, *M. pneumoniae* was usually described to cause mild and self-limiting infections [6]. *M. pneumoniae* has also been suggested to be a common finding in the upper respiratory tract of asymptomatic individuals [11]. In this case series of mycoplasma PCR-positive cases, the patients had quite severe symptoms. In addition to fever and cough, shortness of breath was a common symptom of *M. pneumoniae* infection even in previously healthy young adults. Nasal symptoms were rare.

Data on the clinical picture of *M. pneumoniae* infection, especially in adults, are limited and conflicting. The most common respiratory symptoms of *M. pneumoniae* infection in this series and in the literature are cough and fever [6], and the lack of nasal symptoms found here and in one previous study in adults is an important clinical note [12]. Unexpectedly, the subjective feeling of dyspnoea or shortness of breath was common in our patients. According to the 2012 Cochrane review [13], wheezing is less common in mycoplasma patients compared with other community-acquired pneumonias. However, afterwards, Medjo et al. found wheezing to be typical in mycoplasma infection [14]. Another study consisting of children indicated that symptoms were similar between *M. pneumoniae* and respiratory virus infections [15]. In this series, the clinical picture of *M. pneumoniae* infection differed from that of viral respiratory infections.

About 5% of our patients required intensive care unit admission. This is consistent with the study of Khyory et al. [3], which found that 16% of patients with *M. pneumoniae* infection required intensive care unit admission and highlighted the role of *M. pneumoniae* as a competent pathogen that could cause severe infections. In general, the incidence of pneumonia is the highest among older adults, and underlying illnesses are present in almost 80% of the patients [16]. In this series of *M. pneumoniae* PCR-positive patients, most of the cases presented in previously healthy young adults without specific risk factors, and the symptoms were not affected by underlying diseases. The most common underlying disease in our series was asthma, which was present in 8% of the patients. This rate is almost equal to the prevalence of asthma in the Finnish general population, and thus, previous asthma seems to not be a risk factor for *M. pneumoniae* infection [17].

Several changes to the antimicrobial therapy were needed in most patients, as their condition did not improve until receiving an antibiotic effective against *M. pneumoniae*. The treatment guidelines in Finland do not endorse the use of tetracyclines, macrolides or fluoroquinolones as the first-line treatment for community-acquired pneumonia. Thus, only 10% received an antibiotic effective against *M. pneumoniae* as the first empiric choice. Only six patients were discharged without effective treatment. Most of the patients went through several changes in their medication, which probably postponed their recovery and prolonged their hospital stay. According to the Cochrane review concluded in 2015, there was not enough evidence to make a specific conclusion about the antibiotic treatment of *M. pneumoniae* infection [9]. Placebo-controlled trials have been suggested [9, 18, 19]. However, our results indicated that it might not be ethically acceptable to perform placebo-controlled trials in *M. pneumoniae* infections, at least in a hospital-based setting.

In children at least, about 30–60% of lower respiratory infections have been found to be mixed infections [8, 16, 20, 21]. In our patients, most infections were isolated *M. pneumoniae* infections, and only eight (10%) patients were detected to have a respiratory virus. Moreover, *M. pneumoniae* was the only bacterium found in multiplex PCR. As the childhood general vaccination with conjugated pneumococcal vaccine has decreased the incidence of classical pneumococcal pneumonia, the proportion of *M. pneumoniae* as the aetiology of CAP in children is increasing [20]. This may also gradually occur among adolescents and adults.

The epidemics of *M. pneumoniae* have been shown to occur mostly in late fall [4, 15]. During this study period, no *M. pneumoniae* epidemic occurred in the area. When the 3 study years were summarised, the incidence seemed to have peaked at late fall even outside epidemics.

A major limitation of this study is its retrospective case analysis design. Thus, the indications for the samples were not necessarily consistent throughout the population, which may have affected for example the age distribution in this study. The information collected from the medical records is always incomplete. Some symptoms (i.e. shortness of breath) were recorded as a subjective report from the patients, limiting the reliability of the results. Moreover, this study was hospital-based and did not include patients treated at primary care.

In conclusion, *M. pneumoniae* can cause severe infections that require hospitalisation and an effective antimicrobial treatment. Pneumonia with cough and dyspnoea without nasal symptoms are clinical signs for clinicians to warrant the PCR test for mycoplasma and to consider the empiric antibiotic covering of *M. pneumoniae*.
